# Exploring the aggregation of four functional measures in a population of older adults with joint pain and comorbidity

**DOI:** 10.1186/1471-2318-13-119

**Published:** 2013-11-05

**Authors:** Lotte AH Hermsen, Stephanie S Leone, Martin Smalbrugge, Dirk L Knol, Henriëtte E van der Horst, Joost Dekker

**Affiliations:** 1 Department of General Practice and Elderly Care Medicine and the EMGO Institute for Health and Care Research, VU University Medical Center, Amsterdam, Netherlands; 2 Public Mental Health, Netherlands Institute of Mental Health and Addiction, Utrecht, Netherlands; 3 Department of Epidemiology and Biostatistics and the EMGO Institute for Health and Care Research, VU University Medical Center, Amsterdam, Netherlands; 4 Department of Rehabilitation Medicine and the EMGO Institute for Health and Care Research, VU University Medical Center, Amsterdam, Netherlands

**Keywords:** Bifactor model, Functioning, ICF model, Older adults, Pain

## Abstract

**Background:**

In clinical settings, it is important for health care providers to measure different aspects of functioning in older adults with joint pain and comorbidity. Besides the use of distinct measures, it could also be attractive to have one general measure of functioning that incorporates several distinct measures, but provides one summary score to quantify overall level of functioning, for example for the identification of older adults at risk of poor functional outcome. Therefore, we selected four measures of functioning: Physical Functioning (PF), Activities of Daily Living (ADL), Instrumental Activities of Daily Living (IADL) and participation, and tested the possibility to aggregate these measures into one general measure of functioning.

**Methods:**

A prospective cohort study of older adults (≥65 years) with joint pain and comorbidity provided baseline data (n = 407) consisting of PF (PF subscale, RAND-36; 10 items), ADL (KATZ index; 6 items), IADL (Lawton index; 7 items) and participation (KAP; 6 items). We tested two models with confirmatory factor analysis: first, a bifactor model with all four measures and second, a bifactor model with PF, ADL and IADL and a correlated but distinct subgroup factor for participation. Several model fit indexes and reliability coefficients, such as explained common variance (ECV) and omegas were computed for both models.

**Results:**

The first model fitted the data well, but the reliability analysis indicated multidimensionality and unique information in the subgroup factor participation. The second model showed similar model fits, but better reliability; *ECV* = 0.67, *omega*-*t* = 0.94, low *omega*-*s* = 0.18-0.22 on the subgroup factors and high *omega* of 0.82 on participation, which all were in favour of the second model.

**Conclusions:**

The results indicate that PF, ADL and IADL could be aggregated into one general measure of functioning, whereas participation should be considered as a distinct measure.

## Background

Joint pain is a common symptom in primary care, which often contributes to impaired functioning, especially among older adults [1,2]. In older people, complaints such as joint pain are often present in combination with other chronic diseases [3-5]. The combination of joint pain and other diseases increases the risk of becoming disabled [6], which highlights the importance of providing appropriate care for this group, with early recognition of older adults at risk of poor functional outcome.

The International Classification of Functioning (ICF) model provides a framework to describe normal and abnormal functioning [7]. The domains *activities* and *participation* capture levels of functioning at an individual and societal level, respectively. In clinical settings, it is important for health care providers to measure aspects of functioning that are incorporated in these two ICF domains, as this contributes to optimal management and treatment of joint pain and comorbidity [8]. For the development of prediction models for the early identification of older adults at risk of poor functional outcome, it may be more attractive to use one general measure for functioning. Such a general measure would include various aspects of functioning, but provides one summary score quantifying the overall level of functioning, which subsequently would enable the development of a general prediction model for poor functional outcome, instead of distinct models for each measure. This may facilitate the creation of a more common language in the identification of older adults at risk of poor functional outcome and subsequent follow-up strategies in primary care.

Some researchers already have suggested combining the *activities* and *participation* domains for assessment, because of their interrelatedness [9]. Furthermore, another study provided evidence for the use of a unidimensional measure for both domains, based on selected ICF measures [10]. This indicates that the domains *activities* and *participation* can be aggregated. However, other studies found impairments, activity limitations and participation restrictions to be only moderately related; these studies reported that impaired older adults, with limitations in activities, were often still capable to participate in social activities [11]. Furthermore, literature emphasize the importance to distinguish between the two concepts for empirical testing and the development of management strategies for disability [12,13].

The contradictory findings in literature denote that it remains challenging to find the best approach in how to use the different measures of functioning and how to optimize the identification of older adults at risk of poor functional outcome. Therefore, in the context of a larger research project aiming to develop a prediction model for poor functional outcome, in the present study we took a first step by exploring the possibility to aggregate four functional measures: Physical Functioning (PF), Activities of Daily Living (ADL), Instrumental Activities of Daily Living (IADL) and participation. Such an overall score incorporates all measures, but enables a more general approach in the identification of older adults at risk of poor functional outcome.

## Methods

### Design/ study population

We used baseline data from a prospective cohort study on functional outcome in older adults with joint pain and comorbidity. Details about the study design, selection procedure and methods have been previously published [14]. The Medical Ethics Committee of the VU University Medical Center, Amsterdam, approved the study protocol. Participants were recruited from 22 general practices in the region of Amsterdam and were eligible for participation if they (i) were 65 years or older; (ii) had two or more chronic diseases and (iii) reported joint pain on most days in the past month in at least one of eight joint pain sites: neck, back, shoulder, elbow, wrist/hand, hip, knee and ankle/foot, in a screening questionnaire [14]. Written informed consent was obtained from all participants.

### Measures

***Physical Functioning*** was measured with the 10-item PF subscale of the RAND-36 Item Health Survey (RAND-36) [15], which measures limitations in vigorous activities (PF1), moderate activities (PF2), carrying groceries (PF3), climbing several stairs (PF4), climbing one stair (PF5), bending/kneeling (PF6), walking more than one kilometre (PF7), walking half a kilometre (PF8), walking 100 metres (PF9) and self-care (PF10). Answers are scored on an ordinal 3-point scale (severe limitations, some limitations, no limitations). The scores were coded, summed and transformed into a scale score ranging from 0 (worst score) to 100 (best score).

***ADL*** was measured with a modified version of the KATZ index of Independence in Activities of Daily Living (ADL) [16], which asks if the respondent needs help with bathing (A1), dressing (A2), toileting (A3), continence (A4), getting out of a chair (A5) and eating (A6). Answers were scored as 0 (independent) or 1 (dependent) and the item scores were summed to a total score of 0 (best score) to 6 (worst score).

***IADL*** was measured with the Lawton Instrumental Activities of Daily Living Index (IADL) [16], which asks if the respondent needs help with using the telephone (I1), travelling (I2), doing groceries (I3), preparing a meal (I4), housework (I5), taking medicine (I6) and managing money (I7). Answers were scored as 0 (independent) or 1 (dependent) and the items were summed to a total score of 0 (best score) to 7 (worst score).

***Participation*** was measured with the 11-item Keele Assessment of Participation (KAP) [17], which measures restrictions in mobility inside the home (P1), mobility outside the home (P2), self-care (P3), looking after home (P4), looking after belongings (P5), looking after dependants (P6), interpersonal interactions (P7), managing money (P8) and participation in work (P9), education (P10) and social activities (P11). Responses were rated on a 5-point rating scale (all, most, some, a little, none of the time). In a previous analysis, we explored the dimensionality of the KAP and the use of a categorical scoring, in which the above mentioned response options ‘all’ to ‘none’ were scored as 0, 1, 2, 3, 4 and the sum of the item scores were calculated. Exploratory factor analyses revealed two underlying constructs in the 11 item KAP questionnaire, of which only the domain ‘participation in basic activities’ (KAPd1; 6 items) showed adequate reliability [18]. Based on these results, we decided to include only KAPd1, which includes the items P1, P2, P3, P4, P5 and P7. We used the categorical 0–4 scoring and calculated a total score with a range from 0 (best score) to 24 (worst score).

### Statistical analysis

We only studied participants with complete data. Descriptive statistics were used to describe the baseline characteristics of the sample and prevalence of limitations in PF, ADL, IADL and participation. Participants were classified as limited in PF if they scored below the cut-off score of 61 (pooled mean score in a general older Dutch population; 63–77 years: mean 64.8, 78 year or older: mean 57.3) [15] and limited in (I)ADL if they reported at least one limitation on the (I)ADL items. Based on a median score of 2, we dichotomized the domain participation in basic activities in 0–1 (good participation) and 2–24 (poor participation).

### Bifactor model

**Figure 1:**
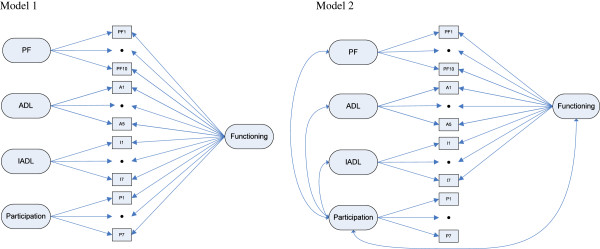
**Illustration of two models.** Model 1: Bifactor model: 1 general factor *functioning* and 4 subgroup factors: physical functioning (PF; 10 items), ADL^*^ (A; 5 items), IADL (I; 7 items) and participation (P; 6 items) Model 2: Combination of a bifactor model: 1 general factor *functioning* and 3 subgroup factors: physical functioning (PF; 10 items), ADL^*^ (A; 5 items), IADL (I; 7 items) and a separate but correlated factor *participation* (P; 6 items) ^*^item A6 (eating) is excluded from the models.

We reversed the PF scoring so that all four functional measures were scored in the same direction, with higher scores reflecting worse functioning. To examine the possibility to aggregate the four functional measures, i.e. physical functioning (PF; 10 items), ADL (A; 6 items), IADL (I; 7 items) and participation (P; 6 items), into one general measure for ‘functioning’ (PF/A/I/P; 29 items), we tested a bifactor model. This model is illustrated in Figure 1, model 1. A bifactor model is an extensive method of confirmatory factor analysis (CFA) [19]. It contains one general factor that is assumed to underlie all items and several subgroup factors that are aspects of this general factor (construct functioning). The general factor and subgroup factors are uncorrelated, which means that the subgroup factors are not dependent on the general factor and thus provide unique information over and above the general factor [20]. In our conceptual model, the bifactor model accounts for (i) the correlations among the 29 items in the general factor ‘functioning’ that reflects the overlap across all items, where higher factor loadings on the general factor indicate that these items contribute more to the general construct ‘functioning’, and (ii) independent subgroup factors, i.e. PF, ADL, IADL, participation, that reflect coherence among specific groups of items and provide unique information over and above the general construct ‘functioning’. In other words, the factor loadings on the subgroup factors provide insight into information that would be lost when aggregating the various measures of functioning, with higher factor loadings on the subgroup factor indicating more loss of information of that item on the aggregated construct [20].

Based on the findings from the above mentioned bifactor model analysis, we subsequently tested a second model that includes a bifactor model, based on three functional measures PF, ADL and IADL, and a separate but correlated factor participation, as illustrated in Figure 1, model 2.

Both models were tested with CFA for ordered categorical items (all 29 individual items are used in their ordinal form), which uses polychoric correlations and the method of weighted least squares with mean and variance adjustment (WLSMV) to estimate the loadings [21]. Factor loadings and residual variance were computed for all individual items. Low residual variance indicated less variation = good item. To evaluate model fits, three absolute close-fit indexes, i.e. ×^2^/df, root mean squared error of approximation (RMSEA) and weighted root mean square residual (WRMSR) and two incremental close-fit indexes, i.e. comparative fit index (CFI) and Tucker-Lewis index (TLI) were used, according to Hu and Bentler [22]. The following cut-off points were considered as indicative of an adequate model fit: ×^2^/df <2, CFI and TLI >0.95, RMSEA <0.06 and WRMSR < 1.0 [22,23].

### Reliability analysis

We computed the proportion of explained common variance (ECV) and the model based reliability coefficients of all included items. The ECV is the ratio of the explained variance by the general factor divided by the variance explained by both the general and the subgroup factors, in which a higher ECV indicates unidimensionality [24]. For reliability assessment, in contrast to the conventional Cronbach’s alpha (which tends to underestimate the reliability of the total score), we calculated model based coefficients *omegas*[25]. This is the preferred method as it assumes no equally sized factor loadings [26]. In order to interpret the *omegas* as sum score reliabilities, we transformed the fitted polychoric correlations back into the original score metric, yielding the fitted covariance matrix and calculated the reliability coefficients based on this matrix, by using a SAS program [27]. This method is preferred, because it is easier to interpret the *omegas* (similar to Cronbach’s alpha). Furthermore, the total score will be applied in clinical practice. Similar to Cronbach’s alpha, *omega*-*total (omega-t)* is an estimator for the reliability of the general factor (total score), thus the proportion of test variance due to all the factors in the model (so items on the general factor and items on the subgroup factors, divided by the error loadings), whereas the *omega-hierarchical (omega-h)* is the proportion of test variance that can be attributed to only the general factor. Next, we calculated the distinct *omegas* for all subgroup factors, in which *subgroup*-*omegas* yield the reliability of the subgroup factors, whereas *omega*-*s* indicates the residual reliability of the subgroup factors [28]. For *omega*-*t*, *omega*-*h* and *subgroup*-*omegas*, a coefficient omega of >0.70 corresponds with an appropriate model, whereas a low *omega*-*s* indicates that the subgroup factor does not contribute to the information provided by *omega*-*t*[28].

## Results

### Sample characteristics

The patient characteristics are presented in Table 1. The study sample consisted of 407 participants, mostly female (62%), with a mean age of 76.8 years (SD = 6.3). Participants often had more than three chronic diseases (48%) and multiple joint complaints (91%). More than half of the participants with at least one functional limitation on the four measures (81%) reported impairment on three or four measures. Complete data on all 29 items were available for 95% of the participants.

**Table 1:** **Sociodemographic and clinical characteristics of the study population** (**N** = **407**)

	
Gender: female, n (%)	254 (62.4)
Age, mean (SD)	76.8 (6.3)
Nationality: Dutch, n (%)	386 (95.1)
Marital status: married/ cohabiting, n (%)	236 (58.7)
Living situation: together, n (%)	242 (59.5)
Educational level, n (%)	
Primary	121 (29.7)
Secondary	199 (48.9)
College/university	87 (21.4)
**Comorbidity**	
Number of chronic diseases, n (%)	
2 chronic diseases	210 (51.6)
≥3 chronic diseases	197 (48.4)
**Joint pain**	
Number of joint pain sites, n (%)	
single	38 (9.4)
multiple	367 (90.6)
Pain duration worst pain site: ≥ 6 months, n (%)	358 (89.5)
Pain intensity CPG (range 0–100), mean (SD)	64.4 (17.3)
**Functional impairment**	
Impaired physical functioning (PF), yes, n (%)	267 (65.6)
ADL limitations (KATZ), yes, n (%)	127 (31.2)
IADL limitations (Lawton), yes, n (%)	248 (60.9)
Participation restriction in basic activities (KAP), yes, n (%)	190 (46.7)
**Number of functional impairments**, n (%)	
1-2	165 (40.6)
3-4	166 (40.7)

*n* = number; *SD* = standard deviation; *CPG* = Chronic Pain Grade; *ADL* = Activities of Daily Living; *IADL* = Instrumental Activities of Daily Living; *KAP* = Keele Assessment of Participation.

### Model 1

At first, we tested a bifactor model that includes all four measures (Figure 1, model 1). The model showed some problems. Firstly, none of the respondents scored positive on item A6: “*Do you need help with eating*?” Therefore, we removed this item from the model. Secondly, the residual variances of item PF8 (walking 0.5 kilometre) and item I1 (using telephone) were negative, which indicated an error in the model. Therefore, we constrained the residual variance to 0.05 for these items. The final adjusted bifactor model fitted the data well. Fit statistics were as follows: ×^2^/df = 534/324 = 1.65, RMSEA = 0.040 (90% CI: 0.034-0.046), CFI = 0.987, TLI = 0.984. Only WRMR showed a minimal deviation compared to the guidelines (1.081).

### Reliability analysis

The ECV (explained common variance) was 0.61, which indicates underlying factors in the general factor, and thus multidimensionality. This was further confirmed when we looked at the *omega*-*s* of the subgroup factors, in which we controlled for the general factor. The results showed values of 0.22, 0.20, 0.15 and 0.53 for PF, ADL, IADL and participation, respectively. In contrast to the first three subgroup factors, the high *omega*-*s* of the subgroup factor participation indicates that the majority of the reliability variance on the participation score is independent of the general factor and thus provides unique information over and above the general factor. This indicates that the subgroup factor participation should be assessed separately.

### Model 2

Based on the above findings, we further tested a combined model, which included a bifactor model with the subgroup factors PF, ADL and IADL and separate but correlated factor participation (Figure 1, model 2). Besides item PF8 and I1 (as seen in model 1), in model 2 we also constrained item A1 to 0.05, because of a negative residual variance. The standardized factor loadings and residual variances for all 29 individual items are presented in Table 2. The fit statistics were similar to model 1: ×^2^/df = 542/327 = 1.65, RMSEA = 0.040 (90% CI: 0.034-0.046), CFI = 0.986, TLI = 0.984, WRMW = 1.083.

**Table 2:** **Standardized factor loadings and reliability coefficients** (**omegas**) **of the most optimal model that combines (i) a bifactor model: general factor and 3 subgroup factors**: **physical functioning** (**PF**; **10 items**), **ADL** (**A**; **6 items**), **IADL** (**I**; **7 items**) **and (ii) a separate but correlated factor participation** (**P**; **6 items**)

	**Bifactor**				**Correlated factor**	**Residual variance**
	**General factor**	**Subgroup factors**				
	**Functioning**	**PF**	**ADL**	**IADL**	**participation**	
PF1 Vigorous activities	0.723	0.046				0.475
PF2 Moderate activities	0.879	0.127				0.211
PF3 Carrying groceries	0.832	0.104				0.298
PF4 Climbing several stairs	0.845	0.346				0.167
PF5 Climbing one stair	0.830	0.384				0.163
PF6 Bending/kneeling	0.581	0.271				0.588
PF7 Walking > 1 kilometre	0.731	0.644				0.076
PF8 Walking 0.5 kilometre	0.625	0.747				0.050†
PF9 Walking 100 metres	0.623	0.755				0.043
PF10 Self-care	0.738	0.161				0.430
A1 Bathing	0.675		0.703			0.050†
A2 Dressing	0.621		0.710			0.110
A3 Toileting	0.608		0.525			0.355
A4 Continence	0.443		−0.016			0.803
A5 Getting out of chair	0.594		0.284			0.566
A6 Eating*	-		-			-
I1 Using telephone	0.384			0.896		0.050†
I2 Travelling	0.742			0.281		0.370
I3 Doing groceries	0.710			0.491		0.254
I4 Preparing meal	0.494			0.433		0.569
I5 Housework	0.747			0.230		0.389
I6 Taking medicine	0.673			0.197		0.508
I7 Managing money	0.397			0.443		0.646
P1 Mobility inside home					0.742	0.450
P2 Mobility outside home					0.876	0.232
P3 Self-care					0.743	0.448
P4 Looking after home					0.723	0.477
P5 Looking after belongings					0.766	0.414
P7 Interpersonal interactions					0.650	0.577
Correlations with the factor participation	0.507	0.229	0.261	0.283	1	
*Omega* (*total and subgroup*)	0.94	0.94	0.50	0.72	0.82	
*Omega hierarchical*	0.79					
*Omega*-*s*		0.22	0.22	0.18		
ECV	0.67					

*Item A6 (eating) is excluded from the bifactor model.

†Constrained to 0.05, because of negative residual variance.

### Reliability analysis

Firstly, we examined the degree to which the general factor was confounded by the subgroup factors. The *omega-t* was 0.94, whereas the *omega-h* was 0.79. This indicates that 0.79/0.94 = 84% of the reliability variance in the total score is due to the general factor for functioning. In other words, the interpretation of the total score was hardly confounded by the subgroup factors. Compared to model 1, the ECV increased to 0.67. The *omega*-*s* of the subgroup factors were all low (0.18 to 0.22), which indicates no unique information on the subgroup factors over and above the general factor. Furthermore, the omega of the subgroup factor participation was 0.82, which indicates good reliability.

## Discussion

In this study, we explored the possibility to aggregate four measures of functioning: Physical Functioning (PF), Activities of Daily Living (ADL), Instrumental Activities of Daily Living (IADL) and participation, into one general measure that quantifies overall level of functioning, by testing a bifactor model. The bifactor model fitted the data well. However, the reliability analysis indicated multidimensionality in the general factor *functioning* and unique information over and above the general factor in the subgroup factor *participation*. Therefore, we tested a second model that included a bifactor model (PF, ADL and IADL) and separate but correlated factor *participation*. Compared to model 1, model 2 showed equal model fits, but better reliability. Thus, the results favour the use of an aggregated measure of PF, ADL and IADL and a separate measure for participation. For research studies, this means that full data should be collected for all four functional measures, but subsequently the researchers can calculate a summary score for PF, ADL and IADL, to assess overall level of functioning and to develop prediction models for poor functional outcome in older populations. Participation should be used as distinct measure.

Two considerations should be made, when interpreting these results. Although the ECV increased in model 2, this increase was lower then expected. This could be explained by the low omega on the subgroup factor ADL (0.50), which indicates that this factor contributes to a lesser extent to the general construct functioning than the other subgroup factors. Thus, one could argue about including ADL as functional outcome in the general measure, as it provides no additional information over and above the measures PF and IADL, at least not in our primary care sample. Furthermore, there is some overlap between the included items within the four measures of functioning. For example, the item ‘self-care’ is assessed in the PF and ADL as well as in the participation measure. One would expect high correlations between these items, which could subsequently be an argument for exclusion of these items. However, we decided to maintain all items in the analyses for two reasons. First, these items are part of existing and validated questionnaires and all necessary to examine the constructs of interest. Second, the conceptual models behind the four measures differ substantially. This can be illustrated by the item ‘self-care’. In the measure PF, participants are asked if they are disabled in *performing* self-care tasks. In the second measure ADL, participants are asked if they *need help* with their self-care. In the third measure participation, participants are asked if they are able to perform their self-care *if and when they want to*, despite possible help from devices or relatives. All three constructs could be related, but this is definitely not always the case. Someone with problems in performing a task does not necessary need help with performing this task. On top of these arguments, a correlation analyses with all items about the example self-care showed correlations between 0.21 and 0.61, which indicates no signs of collinearity (data not shown). This also accounts for other overlapping items in the four measurements.

Until now, there is no consensus about the most optimal use and application of the ICF domains activities and participation to study level of disability in research. In the pre-final version of the ICF model, activities and participation were presented as two separate domains, but in the final version these two domains were again combined into one concept, because of the difficulty to distinguish between the two domains. The WHO stated that despite the combined concept, the two domains still have two distinct definitions and remain distinguishable. According to the ICF model, activity limitations are defined as difficulties in performing a task, whereas participation restrictions are defined as difficulties in engaging in life situations [7]. Several studies have investigated the similarities and overlap between the two domains and found conflicting results. Some studies found evidence for two dimensions [12,29] and suggested separation of the domains when for example analyzing intervention effects, as intervention could have different effects on both domains [30]. These studies highlighted the difficulties in making a distinction between the two domains, as the selected instruments often measured aspects of activities as well as aspects of participation [31-33]. But it has been suggested that these problems may be due to measurement problems, rather than the constructs being intrinsically different. On the other hand, there are also studies that support combining the activity and participation domain [34,35]. All these contradictory findings and the ongoing debate in literature highlight the challenge in the classification and subsequent use of the ICF domains in research. Our findings seem to confirm the classification of the WHO, in which activities and participation are two distinct domains that both provide unique information about the level of functioning.

Our study has several strengths. We used validated questionnaires to measure functioning and had full data available from almost all participants. Also, besides testing the bifactor model, we examined the reliability of the model, by investigating the omegas of both the general factor and subgroup factors. However, some limitations of the study should also be mentioned. Firstly, there are contradictory findings in the literature about the dimensionality of the PF subscale of the RAND-36. As intended, many studies found evidence for the PF subscale to measure a unidimensional construct [36,37]. However, some studies have indicated that this 10-item subscale is multidimensional, with interdependency between the items [38,39]. Based on the moderate support for unidimensionality, its relevant items, its extensive use and its feasibility in practice, we decided to include this questionnaire as a measure for physical functioning. The testing of the models showed no problems with respect to the 10 items of the PF questionnaire and therefore we reasoned that this questionnaire was indeed a suitable measure for this study. Secondly, earlier research suggested that measuring ADL may be more relevant in clinical settings, like hospitals or nursing homes, because of the more extensive problems the residents face [40]. In our study population of older adults, selected in general practices, we found a relatively low prevalence of ADL limitations, which confirmed earlier results [1]. Nevertheless, we decided to include this measure to provide a complete picture of the impact of joint pain and comorbidity on different aspects of functioning.

## Conclusions

The results of our analysis support the use of an aggregated functional measure for PF, ADL and IADL, whereas participation should be considered as a distinct measure, in research studies that have the broader aim to develop prediction models for poor functional outcome.

## Competing interests

The authors declare that they have no competing interests.

## Author’s contributions

LH: Study design; data collection; data analysis; interpretation; manuscript preparation. DLK: data analysis; interpretation, manuscript preparations. SL, MS, JD, HvdH: study design; interpretation; manuscript preparations. LH drafted the article. SL, DLK, MS, JD and HvdH discussed all versions of the manuscript. All authors revised and approved the final version of the manuscript.

## Pre-publication history

The pre-publication history for this paper can be accessed here:

http://www.biomedcentral.com/1471-2318/13/119/prepub
